# Dexmedetomidine ameliorates hepatic ischemia reperfusion injury via modulating SIRT3 mediated mitochondrial quality control

**DOI:** 10.1038/s41598-025-90069-1

**Published:** 2025-02-15

**Authors:** Xiaqing Ning, Jilang Tang, Xueqin Li, Jiaqi Wang, Fan Zhai, Congcong Jiang, Shixia Zhang

**Affiliations:** College of Veterinary Medicine, Hebei Agricultural University, No. 2596 Lekai South Street, Lianchi District, Baoding, 071000 People’s Republic of China

**Keywords:** Dexmedetomidine, Ischemia-reperfusion, Liver, Mitochondrial biogenesis, Mitochondrial dynamics, SIRT3, Haematological diseases, Biochemistry

## Abstract

Ischaemia-reperfusion (IR) damage is an inevitable adverse effect of liver surgery. Recent research has found that IR damage is involved in severe mitochondrial dysfunction. Mitochondrial biosynthesis and dynamics control mitochondrial mass, distribution, and function. Sirtuin 3 (SIRT3) is widely known for preserving health and functionality of mitochondria. DEX has been proven to alleviate liver damage through antioxidant and anti-apoptotic pathways. But it’s unclear how DEX protects mitochondria at this time. In this research, the mechanism behind the protective benefits of DEX was examined using the rat liver IR model and the rat liver cells (BRL-3 A) hypoxia reoxygenation (HR) model. We discovered that DEX treatment restored mitochondrial membrane potential, promoted ATP production, prevented oxidative stress, and decreased apoptosis in BRL-3 A cells. Furthermore, HR damage increased mitochondrial fission while decreasing mitochondrial fusion and biogenesis in BRL-3 A cells, which DEX partially corrected. The benefits of DEX on mitochondrial protection were reversed after addition of SR-18,292. Additionally, DEX showed the ability to enhance SIRT3 expression, and after cells were transfected with SIRT3 siRNA, DEX’s effects on mitochondria were partially prevented. Similarly, in the rat model, DEX alleviating liver histopathological injury and oxidative stress. DEX inhibited IR-induced mitochondrial damage through improving ETC complex I- IV activities and ATP content, reducing apoptosis, controlling mitochondrial quality, and upregulating the expression of SIRT3. Additionally, our research shows that DEX’s ability to protect the liver against IR damage is mediated by the modulation of mitochondrial quality control. Overall, the modification of SIRT3 activity could be responsible for this outcome.

## Introduction

A risk factor for partial hepatectomy and liver transplantation is hepatic ischemia-reperfusion (IR) injury^1,2^. At the moment, reducing IR injury represents a significant clinical problem. Although there are some potential measures that have shown promise, it is vital to create new hepatoprotective techniques to combat this life-threatening condition^3,4^. Liver IR injury is a very complicated pathophysiological process that includes oxidative stress, apoptosis, and mitochondrial dysfunction^5^. Animal studies have provided some prospective therapeutic approaches for liver IR injury, the precise processes remain a mystery. Investigating the molecular processes underlying the onset of liver IR injury and taking into account potential therapeutic approaches are essential to reducing mortality brought on by IR injury.

One important organ that is vulnerable to harm from oxidative stress is the liver^6^. Apoptosis and the degradation of liver morphology were caused by increased oxidative stress^7,8^. IR injury is also confirmed to have oxidative stress as a significant etiology in the meantime^9,10^. Cellular bio-oxidation is integrated by mitochondria and produces 95% of the adenosine triphosphate (ATP) needed to support the many functions of the liver. When ROS are created in excess, ATP synthesis fails, Cytochrome c is released, and cells suffer irreparable damage or die^11^. In order to lessen liver IR damage, it may be necessary to limit oxidative stress and reduce the ensuing mitochondrial malfunction.

Nuclear respiratory factor 1 (Nrf1), peroxisome proliferator-activated receptor coactivator-1 (PGC-1), and mitochondrial transcription factor A (TFAM) are all involved in the biogenesis of mitochondria^12^. Impaired mitochondrial biogenesis can exacerbate mitochondrial dysfunction. Fusion and fission homeostasis are essential for controlling mitochondrial dynamics. Mitofusin (MFN) 1 and 2 and the optic atrophy 1 (OPA1) protein are engaged in mitochondrial fusion, whereas dynamin-related protein 1 (DRP1) and fission protein 1 (Fis1) are involved in mitochondrial fission^13,14^. Lipid peroxidation, increased ROS production, inhibition of respiration, and decreased ATP synthesis were all consequences of the mitochondria’s imbalance between fusion and fission^15^. Toyama et al. described that hepatic IR resulted in a lack of ATP production, which increased the expression both DRP1 and Fis1^16^. Then again, hepatic IR dramatically decreases mitochondrial biogenesis^17^. A study in the cultured hepatocyte hypoxia-reoxygenation (HR) model has shown that mitochondrial mass and potential for membranes increased owing to accelerated mitochondrial biogenesis^18^. Pretreatment with a mitochondrial fission inhibitor can also protect the cerebrum from IR destruction by lowering oxygen consumption, regulating ROS generation, and decreasing cell death^19^. However, to comprehend the effects of hepatic IR on the mitochondria and their regulatory signals, further thorough investigations are required.

In the mitochondrial matrix of liver cells, sirtuin 3 (SIRT3) is a sirtuin that is abundantly expressed^20^. Intact SIRT3 is found in the cytoplasm and nucleus and is cleaved into short-chain forms after translocation to mitochondria. Through its deacetylation, SIRT3 controls a number of biological processes within the cell, such as metabolic dynamics, the breakdown of energy^21^. SIRT3’s necessity for maintaining mitochondrial homeostasis is therefore logical. Various experimental models of IR injury have shown beneficial effects of regulating SIRT3 expression on tissue injury^22,23^. Therefore, the therapeutic strategy of regulating SIRT3 expression may be an effective method of mediating mitochondrial quality control and liver protection during IR injury.

Due to its anxiolytic and calming effects without affecting breathing and with only moderate side effects, intensive care units(ICUs) and medical anesthesia have both made substantial use of dexmedetomidine (DEX). In recent years its potential organ protective effects has become a major research direction^24^. DEX protects hepatocytes toward HR injury, according to Zhao et al., by lowering oxidative stress and apoptosis^25^. By reducing alterations in mitochondrial fusion and fission, DEX also has strong cytoprotective benefits against Cecal Ligation and Puncture (CLP)-induced lung damage^26^. DEX may also reduce renal ischemia-reperfusion injury in rats by inhibiting the opening of mitochondrial permeability transition pores through SIRT3-mediated deacetylation of cyclin D, protecting mitochondria, and reducing damage^27^. However, the precise mechanism underlying DEX’s hepatoprotective effects is still poorly known.

Liver IR injury is difficult to avoid in surgeries such as liver resection and liver transplantation, which can cause liver failure and even damage the function of distant organs, seriously affecting the prognosis of the body. It is particularly important to explore effective methods to prevent or alleviate Liver IR injury. In order to explore the specific protective mechanism of DEX in Liver IR injury, we established in vivo and in vitro models to test the defense mechanisms of DEX against liver IR harm. Our research centered on the significant advantages of DEX in reducing mitochondrial damage and controlling mitochondrial quality, and clarified the possibility that the SIRT3 pathway may play a role in how DEX exerts its protective properties.

## Materials and methods

### Cell model and grouping

BRL-3 A cells (Hunan Fenghui Biotechnology Co., Ltd., Hunan, China) were raised in DMEM, a Dulbecco’s modified Eagle’s medium, supplemented 10% fetal bovine serum (FBS, Solarbio, Beijing, China), 1% streptomycin, and 1% penicillin (Solarbio, Beijing, China). The cells were cultured at 37℃ with 5% CO_2_. When cells were about 80% confluent, the cells underwent passage.

BRL-3 A cells were used to create an HR model to imitate the liver IR injury previously observed^28^. The cells were first treated for 1 h with 5 mmol/L Na_2_S_2_O_4_ (Beijing Bailingwei Technology Co., Ltd., Beijing, China), after which the culture media was removed, the cells were hypoxic, and then they were reoxygenated for 2 h with DMEM.

#### Group design

The following ten groups were randomly selected from the cell model: Control group: the cells were given DMEM treatment, HR group: the cells were subjected to the HR conditions, DEX group: the cells were given 10 µmol/L DEX (Shanghai Yuanye Biotechnology Co., Ltd., Shanghai, China) 2 h^29^, DEX + HR group: the cells were administered 10 µmol/L DEX 2 h and then subjected to the HR conditions, SR-18,292 + DEX + HR group: cells received 20 µmol/L SR-18,292 (Sigma Aldrich, USA) within 18 h, followed by 10 µmol/L DEX with 2 h, and finally HR conditions^30^, SR-18,292 + HR group: the cells were given treatment 20 µmol/L SR-18,292 with 18 h and then exposed to the HR conditions, Mdivi-1 + DEX + HR group: cells received 25 µmol/L Mdivi-1 (Macklin, China) within 24 h, followed by 10 µmol/L DEX with 2 h, and finally HR conditions^31^, Mdivi-1 + HR group: the cells were given treatment 25 µmol/L Mdivi-1 with 24 h and then exposed to the HR conditions, si-SIRT3 + DEX + HR group: prior to being subjected to the HR conditions, the cells were pre-transfected with SIRT3 siRNA and given the DEX treatment, si-SIRT3 + HR group: SIRT3 siRNA was pre-transfected into the cells and then subjected to the HR conditions. Then the cells were lysed, RNA and protein were collected for further measurement.

#### Small interfering RNAs (siRNAs) transfection

GenePharma (Shanghai, China) generated si-SIRT3_1, si-SIRT3_2, or negative control (si-NC), which were then transfected into BRL-3 A cells using Lipofectamine 2000 (Invitrogen, Darmstadt, Germany) in accordance with the manufacturer’s instructions. Table 1 lists the sequences for the negative control and si-SIRT3.

**Table 1:** The sequences for the negative control and si-SIRT3.

Gene	Forward(5′ − 3′)	Reverse(5′- 3′)
si-NC	GGCACAGTCAAGGCTGAGAATG	ATGGTGGTGAAGACGCCAGTA
si-SIRT3_1	CCAGUGGUAUCCCAGACUUTT	AAGUCUGGGAUACCACUGGTT
si-SIRT3_2	CCAAUGUCGCUCACUACUUTT	AAGUAGUGAGCGACAUUGGTT

### Animal model and grouping

SPF Biotechnology Co., Ltd. (Beijing, China) provided the 30 male SD rats (weight 180–220 g) used in this investigation. All rats were maintained at a temperature of roughly 20℃ with 12-hour light-dark cycles after being acclimated to the lab for at least seven days. 12 h before to surgery, all food was withdrawn, but the rats were given water. Every single animal experiment was conducted in conformity with the standards and laws established by Hebei Agriculture University’s institutional animal care and use committee (2022029). The study was carried out in compliance with the ARRIVE guidelines.

#### Hepatic IR model

A 70% Hepatic IR model was run in accordance with earlier findings^32^. The animals were kept anesthetized using a cocktail of ketamine and xylazine for induction, followed by isoflurane for maintenance during surgery. In order to induce ischemia in the left and center hepatic lobes, the portal arteries were clipped with an atraumatic device. Tissue blanching demonstrated the presence of ischemia. The clamp was taken off after 30 min of ischemia to allow for reperfusion. The quick color change of the ischemic lobes following clamp release served as proof of reperfusion.

#### Group design

20, 50 and 100 µg/kg of DEX were given 30 min before to ischemia. A DEX dose of 50 µg/kg was chosen as the ideal dose for additional biochemical tests based on serum ALT activity. Rats (*n* = 6) were randomly assigned to one of three groups in the current study: the sham group, the IR group, or the DEX + IR group. On the sham group, a laparotomy and portal triad exposure without occlusion were carried out. The IR group underwent laparotomy, portal triad exposure with occlusion, and no medication was used. The DEX + IR group received a DEX injection 30 min before to the hepatic lobes’ ischemia. Rats were euthanized by deep inhalation of isoflurane six hours after reperfusion. Serum was extracted from blood samples and kept at -80℃ until use. Quickly removed from the liver tissues, and one portion of the middle liver lobe was fixed in 2.5% glutaraldehyde fixative solution and the other portion was imbedded in 10% formalin solution. To facilitate additional research, the left lobe was kept at -80℃.

### Assay for cell viability

The CCK-8 test (Dojindo, Kumamoto, Japan) was utilized to determine the vitality of BRL-3 A cells in accordance with the manufacturer’s recommendations. In a nutshell, once the groups had their particular treatments, the cells were digested and planted at a density of 2500 cells per well on 96-well plates. Following that, 100 µL of DMEM supplemented with 10 µL of CCK-8 solution were added to the 96-well microtiter plate, which was then incubated at 37 °C for 1 h. The absorbance of each well was determined at 450 nm using a multi-plate reader (BioTEK, United States).

### Level of intracellular reactive oxygen species (ROS)

Using the previously published DCFH-DA (Invitrogen) method, intracellular ROS levels were discovered^33^. Briefly, after being rinsed three times with pre-cooled PBS, BRL-3 A cells in the designated groups were treated with DCFH-DA (10 µmol/L) at 37 °C in the dark for 15 min. The fluorescence was measured using a fluorescence microplate reader (Synergy2, BioTek Instruments, Inc.). The ROS levels were calculated using the mean fluorescence intensity per cell.

### Biochemical assays

Using commercial assay kits (Nanjing Jiancheng, China), we measured the levels of GSH-PX, T-AOC, H_2_O_2_, and NO in BRL-3 A cells as well as the levels of MDA, SOD, and CAT in liver tissues in accordance with the manufacturer’s instructions. Using a UniCel DxC800 Synchron chemistry system (Beckman, USA), serum ALT activities were assessed.

### Observation of liver morphology

#### Liver histopathology

The liver tissues were dehydrated using an alcohol series, cleared with toluene, and contained within paraffin. The tissues were then divided into 5 μm-thick sections, stained with haematoxylin and eosin (H&E), and analyzed histologically under a light microscope. Liver injury was graded on a scale of 0 to 4 for congestion, vacuolization, and necrosis^34^.

#### Liver ultrastructure

Razor blades were used to slice the fresh liver tissues into 1 mm^3^ pieces, which were subsequently pre-fixed in glutaraldehyde (2.5%), followed by three PBS washes. The tissues were subsequently post-fixed with 1% osmic acid for a period of 2 h. They were then implanted in epoxy resin and dehydrated using gradient ethanol. The implanted tissues were subsequently separated into 50 nm-wide ultrathin sections. Uranyl acetate and lead citrate were used as additional stains on these portions. A transmission electron microscope (TEM; H-7650, Hitachi, Japan) was used to view the stained slices.From each group, three animals were chosen at random for ultrastructural analysis.

### Measurement of respiratory chain complexes I - IV activities

Following the instructions, the mitochondria were isolated from fresh liver tissues of each group. Then, utilizing mitochondrial respiratory chain complexes from micro mitochondria Kits for measuring I - IV activity (Solarbio, China), the mitochondria were diluted properly to determine complexes I - IV activities. For complexes I, II, III, and IV, respectively, the absorbance was determined using a visible spectrophotometer (Bejing Purkinje General Instrument Co., Ltd., China) at 340, 605, 550, and 550 nm.

### Determination of ATP content in cells and liver

The ATP Assay Kit (Beyotime, China) was used to measure the amount of ATP present in the liver tissue and BRL-3 A cells. The fresh liver tissue (0.1 g) and cells were centrifuged at 12,000 g for 5 min at 4 °C after being lysed by adding lysis solution as directed. After that, get the supernatant. combining the working solution for ATP determination with the sample to determine the ATP content.

### Mitochondrial membrane potential (MMP) assay

An MMP assay kit with JC-1 (Beyotime, China) was used to measure MMP. In a nutshell, JC-1 staining solution was incubated with BRL-3 A cells for 20 min at 37 °C and in the dark. After incubation, followed by washes with JC-1 staining buffer (1X) for two times. The intensity of the green and red fluorescence was examined using a fluorescence microscope.

### Measurement of tricarboxylic acid (TCA) cycle enzyme activities

Using commercial kits from Solarbio, we measured the activities of the TCA cycle enzyme, including pyruvate dehydrogenase (PDH), α-ketoglutarate dehydrogenase (α-KGDH), succinic dehydrogenase, and isocitric dehydrogenase (ICDHm). The liver samples were first defrosted at 4 °C, and then homogenized (1 g tissue in 9 mL PBS) in pH 7.4 phosphate-buffered saline. In order to find PDH, α-KGDH, and SDH activity, the supernatant was employed. To find ICDHm activity, the mitochondria were separated from the liver tissues using differential centrifugation.

### Apoptosis assay

BRL-3 A cells’ apoptosis was assessed using the Annexin V-fluorescein isothiocyanate (FITC)/prodium iodide (PI) apoptosis detection kit (Wanleibio, China). In a nutshell, cells were seeded into 24-well plates, after apoptosis treatment the wells were subsequently given PBS washes two times. The prepared apoptosis staining solution was then applied to each well at a volume of 250 µL. Incubate the cells at room temperature for 15 min while avoiding light, and then examine the findings with a fluorescence microscope.

### Liver and cell real-time PCR analysis

Using the TRIzol reagent (TransGen Biotech, Beijing, China), total RNA from cultured hepatocytes and left liver lobe tissues was extracted. The PrieScriptTM RT Master Mix (TaKaRa Bio, Dalian, China) was then used to reverse-transcribe the total RNA into cDNA. Sangon Biotech Co., Ltd. (Shanghai, China) created and synthesized the primers indicated in (Table 2). TB Green^®^Premix Ex TaqTMII (TaKaRa Bio, Dalian, China) and the LightCycler^®^ 96 real-time PCR equipment (Roche, Switzerland) were used to measure the amounts of mRNA. The 2 ^−ΔΔCt^ approach was used to examine the relative expression, which was expressed as a function of the threshold cycle (Ct).

**Table 2:** Primer sequence of the genes were tested in the present study.

Gene	Forward(5′ − 3′)	Reverse(5′- 3′)
Gapdh	GGCACAGTCAAGGCTGAGAATG	ATGGTGGTGAAGACGCCAGTA
Nrf1	TCTGCTGTGGCTGATGGAGAGG	GATGCTTGCGTCGTCTGGATGG
TFAM	GTGATCTCATCCGTCGCAGTGTG	TGCCCAATCCCAATGACAACTCTG
PGC-1α	CCACTACAGACACCGCACACATC	GTATTCGTCCCTCTTGAGCCTTTCG
Bcl-2	GAGACACGGCTGCCAGGAC	GCGACGGTAGCGACGAGAG
BAX	GGAGACACCTGAGCTGACCTTG	CATCGCCAATTCGCCTGAGAC
Cleaved Caspase-3	CGGTATTGAGACAGACAGTGGAAC	GCGGTAGAGTAAGCATACAGGAAG
SIRT3	TCAGCAGTATGACATCCCGTACCC	CGTGAAGCAGCCGAAGGAAGTAG

### Liver and cell western blot analysis

Western blotting was used to determine the expression of the proteins Cleaved Caspase-3, BAX, Bcl-2, OPA1, MFN2, DRP1, Cytochrome c, and SIRT3. The internal reference was β-actin. Nuclear and cytoplasmic extraction reagents were used to extract nuclear and cytoplasmic proteins in accordance with the manufacturer’s instructions (Solarbio, China). Following their separation, these proteins were transferred to polyvinylidene fluoride (PVDF) membranes using 10% sodium dodecyl sulfate polyacrylamide gel electrophoresis (SDS-PAGE). After two hours of blocking with 5% skim milk, the membranes were incubated with rabbit anti-Cleaved Caspase-3 (1:500; Wanleibio, China), rabbit anti-BAX (1:500; Wanleibio, China), rabbit anti-Bcl-2 (1:500; Wanleibio, China), rabbit anti-OPA1 (1:1,000; Proteintech, China), rabbit anti-MFN2 (1:2, 000; Proteintech, China), rabbit anti-DRP1 (1:1500; Wanleibio, China), rabbit anti-Cytochrome c (1:1000; Wanleibio, China), rabbit anti-SIRT3 (1:1000; Wanleibio, China) and β-actin (1:500; Bioss, China). The membranes were cleaned with TBST solution before being treated for 2 h with secondary antibodies that were alkaline phosphatase-labeled (1:500; Bioss, China). The bands were found using an Imager Amersham 600 chemiluminescence system (General Electric Company, Little CT, USA), and analyzed gray level by Image J software.

### Statistical analysis

The statistical analysis program SPSS 22.0 (SPSS, IL, USA) was used to examine the data, which were presented as mean standard deviation (X ± SD). Oneway ANOVA followed by Tukey’s post hoc test was used to assess differences between groups for statistical significance; *P* < 0.05 was used to evaluate statistical significance. Graphs were created using Graphpad Prism 9 (San Diego, California).

## Results

### Effect of DEX on cell viability with or without hypoxia reoxygenation injury

**Fig. 1:**
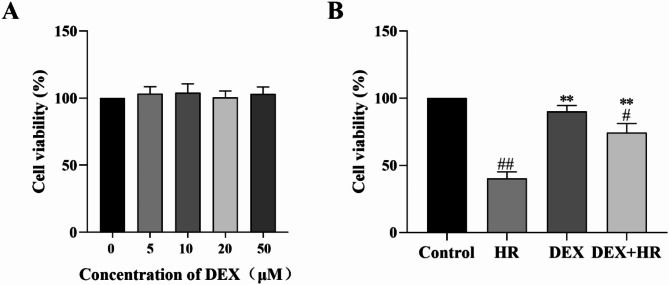
DEX’s impact on the feasibility of BRL-3 A. All data are presented as mean ± SD (*n* = 3). Compared with the control group, ^##^*p* < 0.01; ^#^*p* < 0.05; compared with the HR group, ^**^p < 0.01.

Employing the CCK-8 method, determine how DEX affects cell viability after HR damage. As demonstrated in (Fig. 1A), no significant changes in cell survival were observed after treat different concentrations of DEX. After DEX preprocessing, there was no discernible difference in the DEX group’s cell viability compared to the control group (*P* > 0.05), but there was a marked decrease in cell viability in the HR group (*P* < 0.01). Nevertheless, the DEX + HR group dramatically improved cell viability relative to the HR group (*P* < 0.01) (Fig. 1B). The findings indicate that DEX can improve cell viability after HR injury.

### DEX protected BRL-3 a cells against hypoxia reoxygenation-induced oxidative damage

**Fig. 2:**
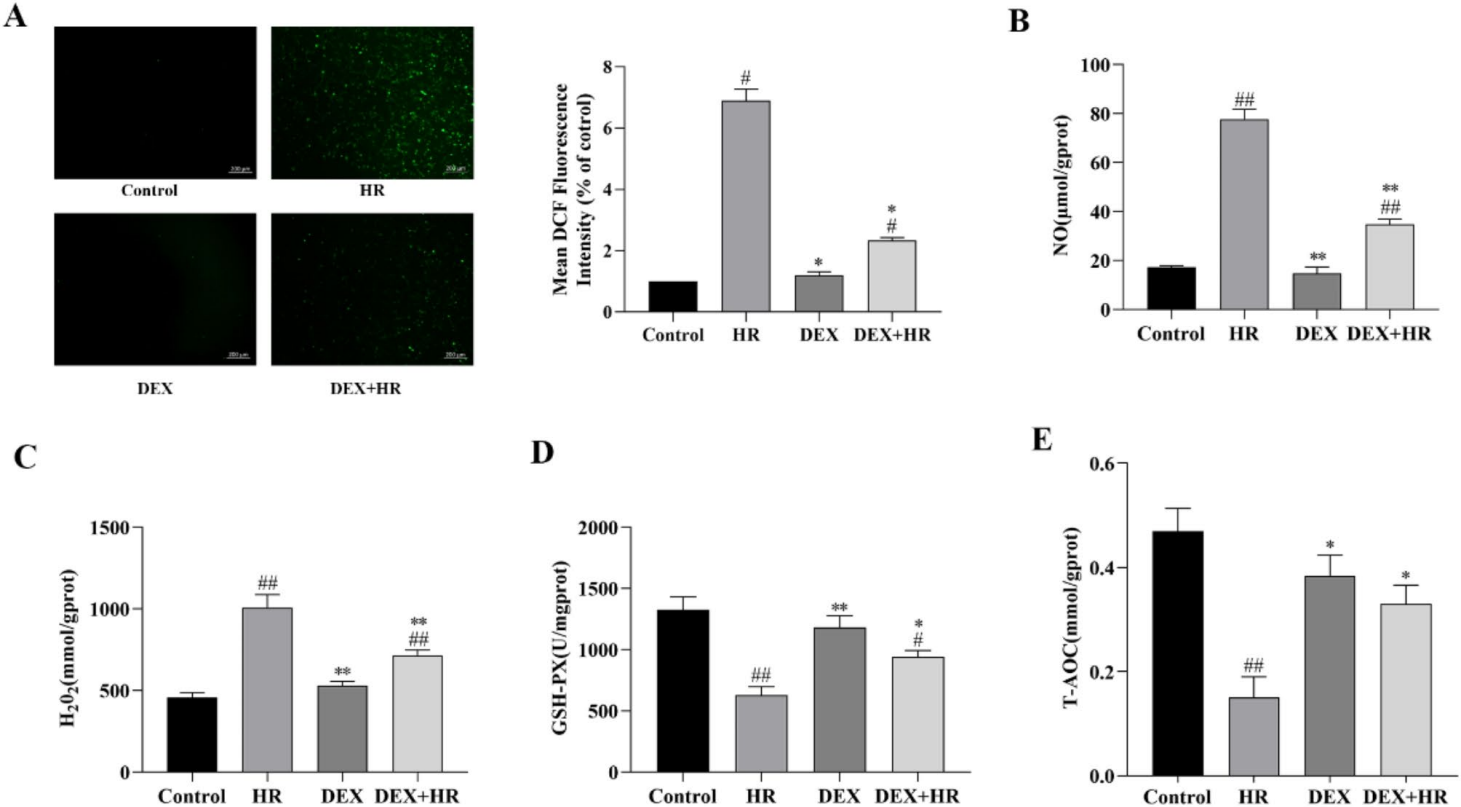
In BRL-3 A cells, DEX reduces oxidative damage brought on by hypoxia reoxygenation. (**A**) DEX prevented the rise in ROS caused by HR, as seen by DCFH-DA fluorescent staining. (**B**–**E**) The oxidative activity indexes that HR elicited in BRL-3 A cells were reversed by DEX. All data are presented as mean ± SD (*n* = 3). Compared with the control group, ^##^*p* < 0.01; ^#^*p* < 0.05; compared with the HR group, ^**^p < 0.01, ^*^p < 0.05.

We assessed ROS, NO, H_2_O_2_, GSH-PX, and T-AOC levels in hypoxic reoxygenated cells to see how DEX affected oxidative stress. As demonstrated in (Fig. 2A–E), Regarding ROS, NO, H_2_O_2_, GSH-PX or T-AOC levels, the DEX group and control group did not differ significantly from one another (*P* > 0.05), HR markedly elevated ROS, NO, and H_2_O_2_ levels (*P* < 0.05 or *P* < 0.01), while decreased the levels of GSH-PX and T-AOC (*P* < 0.01). However, the ROS, NO, and H_2_O_2_ concentrations in the DEX + HR group were lower than those in the HR group (*P* < 0.05 or *P* < 0.01), but GSH-PX and T-AOC levels rose (*P* < 0.05). These results imply that DEX lessens the oxidative stress induced by HR damage.

### DEX protected BRL-3 a cells against hypoxia reoxygenation-induced mitochondria dysfuction and mitochondria-dependent apoptosis

**Fig. 3:**
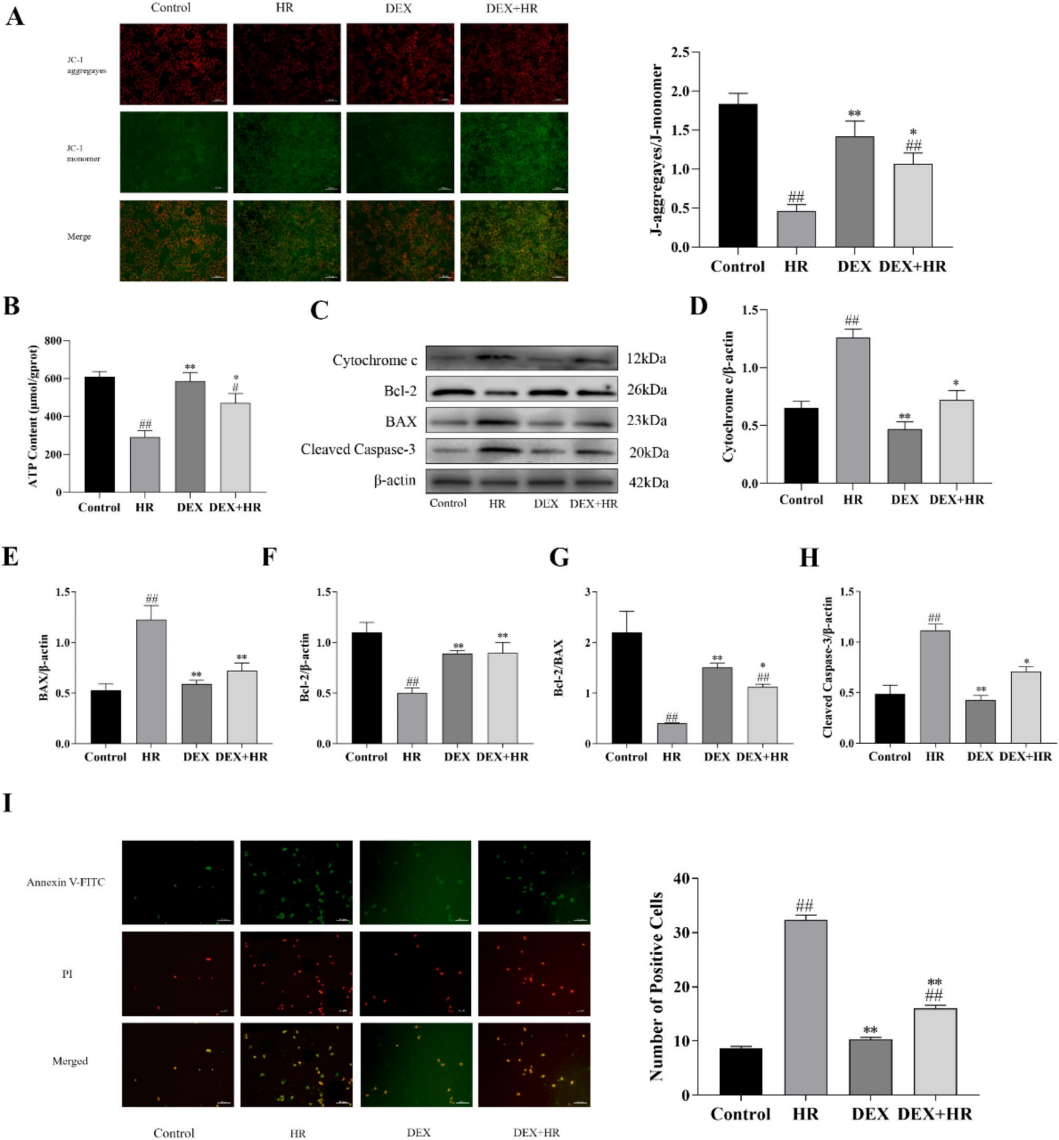
DEX inhibits mitochondrial dysfunction and apoptosis induced by hypoxia reoxygenation in BRL-3 A cells. (**A**) Mitochondrial membrane potential test using JC-1 in BRL-3 A cells (scale bar = 50 μm), quantitative analysis represented by the ratio of JC-1 monomers to aggregates (red fluorescence to green fluorescence). (**B**) The proportional amount of ATP found in BRL-3 A cells. (**C**–**H**) The impact of DEX on the proteins linked to apoptosis that HR induces. The expression of Cytochrome c, Cleaved caspase-3, Bcl-2, and BAX proteins was evaluated by Western blotting. (**I**) By staining with Annexin V-FITC and PI, apoptosis was discovered. (scale bar = 200 μm). All data are presented as mean ± SD (*n* = 3). Compared with the control group, ^##^*p* < 0.01, ^#^*p* < 0.05; compared with the HR group, ^**^p < 0.01, ^*^p < 0.05.

We examined the MMP, mitochondrial ATP level, and mitochondria-dependent apoptosis level to evaluate the effect of DEX on the functioning of mitochondria of BRL-3A cells damaged by HR. In BRL-3A cells, HR caused a substantial reduction in mitochondrial membrane potential (*P* < 0.01), as shown by a decrease in JC-1 aggregates (red fluorescence) and a rise in JC-1 monomers (green fluorescence) (Fig. 3A). The amount of ATP necessary for cellular physiological processes was also significantly lowered (*P* < 0.01) (Fig. 3B). DEX significantly restored the reductions in the potential of mitochondrial membranes and ATP (*P* < 0.05). Additionally, the levels of the proteins Cytochrome c, Cleaved caspase-3, and BAX were greater in the HR group than they were in the control group (*P* < 0.01), nonetheless, the DEX + HR group had lesser results than the HR group did (*P* < 0.05 or *P* < 0.01). Conversely, Bcl-2 and Bcl-2/BAX protein levels were lower in the HR group than they were in the control group (*P* < 0.01), but was higher in the DEX + HR group than in the HR group (*P* < 0.05 or *P* < 0.01) (Fig. 3C–H). Additionally, as displayed in (Fig. 3I), comparing to the control group, the HR group displayed a substantial increase in the amount of apoptotic cells (*P* < 0.01), while after DEX therapy, the quantity of apoptotic cells decreased (*P* < 0.01). These results imply that DEX alleviates mitochondrial dysfunction and mitochondrial dependent cell apoptosis.

### DEX reduce mitochondrial damage is related to mitochondrial quality control in BRL-3 a cells after hypoxia reoxygenation insult

**Fig. 4:**
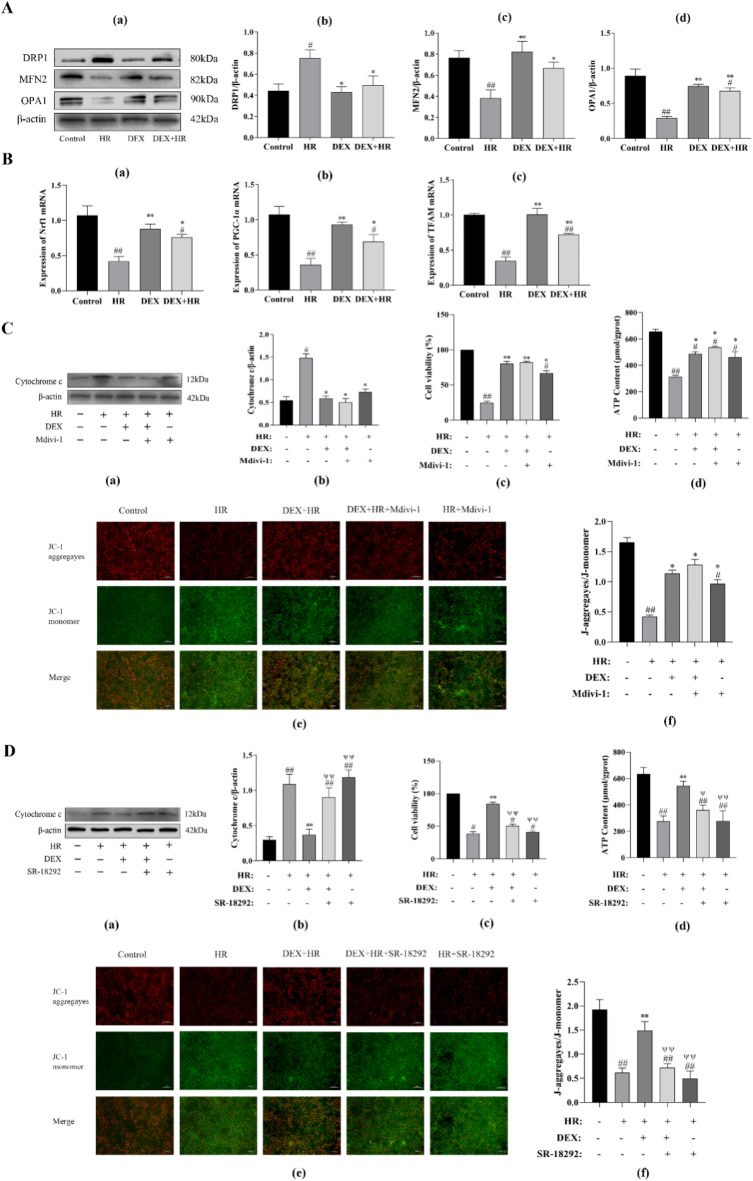
After an HR insult, DEX controls the mitochondrial quality control mechanism in BRL-3 A cells to decrease mitochondrial damage. (**A**) The impact of DEX on proteins relevant to mitochondrial kinetics caused by HR. (**a**) Representative blots. (**b**) DRP1 expression. (**c**) MFN2 expression. (**d**) OPA1 expression. (**B**) The expression of proteins associated to mitochondrial biogenesis is decreased by DEX. mRNA expression of Nrf1 (**a**), PGC-1 (**b**), and TFAM (**c**) was assessed by RT-PCR. (**C**) DEX reduce mitochondrial damage by inhibiting mitochondrial fission. (**a**) The impact of DEX on mdivi-1-induced apoptosis in BRL-3 A cells were evaluated by using Western-blotting technique. (**b**) The expression of cytochrome C. (**c**) The impact of mdivi-1 and DEX addition on BRL-3 A activity. (**d**) The proportional amount of ATP found in BRL-3 A cells. (**e**) In BRL-3 A cells, the mitochondrial membrane potential was measured using the JC-1 assay (scale bar: 50 μm). (**f**) The quantitative analysis was reported as the ratio of JC-1 aggregates to monomers. (**D**) DEX reduce mitochondrial damage by promoting mitochondrial biogenesis. (**a**) The impact of DEX on SR-18,292-induced apoptosis in BRL-3 A cells were evaluated by using Western-blotting technique. (**b**) The expression of cytochrome C. (**c**) The impact of adding SR-18,292 on the activity of BRL-3 A by DEX. (**d**) The proportional amount of ATP found in BRL-3 A cells. (**e**) In BRL-3 A cells, the mitochondrial membrane potential was measured using the JC-1 assay (scale bar: 50 μm). (**f**) The quantitative analysis was reported as the ratio of JC-1 aggregates to monomers. All data are presented as mean ± SD (*n* = 3). Compared with the control group, ^##^*p* < 0.01, ^#^*p* < 0.05; compared with the HR group, ^**^p < 0.01, ^*^p < 0.05.

We measured the expression of proteins linked to mitochondrial dynamics and the mRNA expression of mitochondrial biogenesis-related genes to investigate DEX’s effects on the mitochondrial quality control system’s regulatory mechanisms. The maintenance of mitochondrial dynamics depends in large part on mitochondrial fission and fusion. We discovered that liver HR damage boosted the protein’s expression linked to mitochondrial fission, DRP1, and decreased the expression of the proteins related to mitochondrial fusion, MFN2 and OPA1 (*P* < 0.05 or *P* < 0.01). By upregulating MFN2, OPA1, and downregulating DRP1 (*P* < 0.05 or *P* < 0.01), DEX facilitated mitochondrial fusion (Fig. 4A). Next, we found that liver HR injury inhibited mitochondrial biogenesis, as evidenced by a drop the expression of mitochondrial biogenesis associated Nrf1, PGC-1α and TFAM (*P* < 0.01). DEX raises Nrf1, PGC-1α and TFAM levels, which drives mitochondrial biogenesis (*P* < 0.05 or *P* < 0.01) (Fig. 4B).

To determine if DEX protects mitochondrial damage and is connected to the mitochondrial quality control system, we added the mitochondrial fission inhibitor mdivi-1 and the mitochondrial biogenesis inhibitor SR-18,292 to detect the changes in cell viability, ATP level, the expression of cytochrome c and membrane potential level respectively. Figure 4C shows the results, the HR group had considerably higher levels of cytochrome c expression (*P* < 0.05), and cell viability, ATP levels and membrane potential levels were reduced (*P* < 0.01), but the effect of HR on these indicators was reversed after the inclusion of Mdivi-1, a mitochondrial division inhibitor, which was comparable to the effect of DEX; According to Fig. 4D, in comparison to the HR group, DEX decreased Cytochrome c expression and increased cell viability, ATP levels and membrane potential levels (*P* < 0.01), whereas following the injection of the inhibitor of mitochondrial biogenesis SR-18,292, DEX had the opposite effect on these indicators (*P* < 0.05 or *P* < 0.01). These findings imply that DEX controls the mitochondrial quality control mechanism, preserving mitochondrial homeostasis, to lessen mitochondrial damage.

### DEX exerts protective effect on BRL-3 a cells after hypoxia reoxygenation insult through regulating the expression of SIRT3

**Fig. 5:**
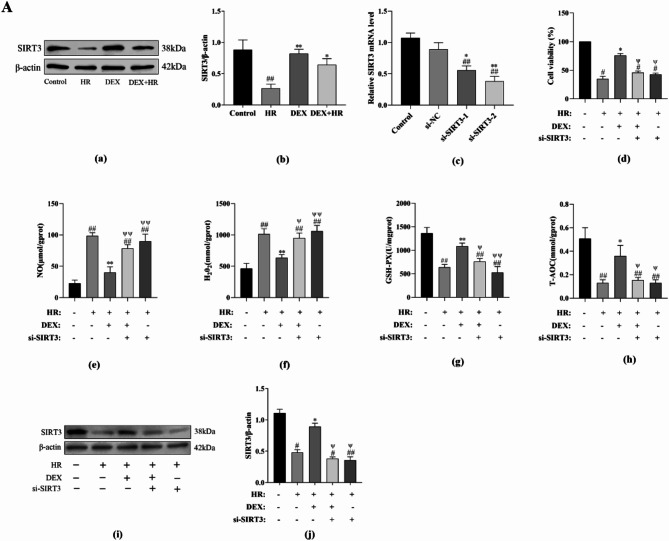

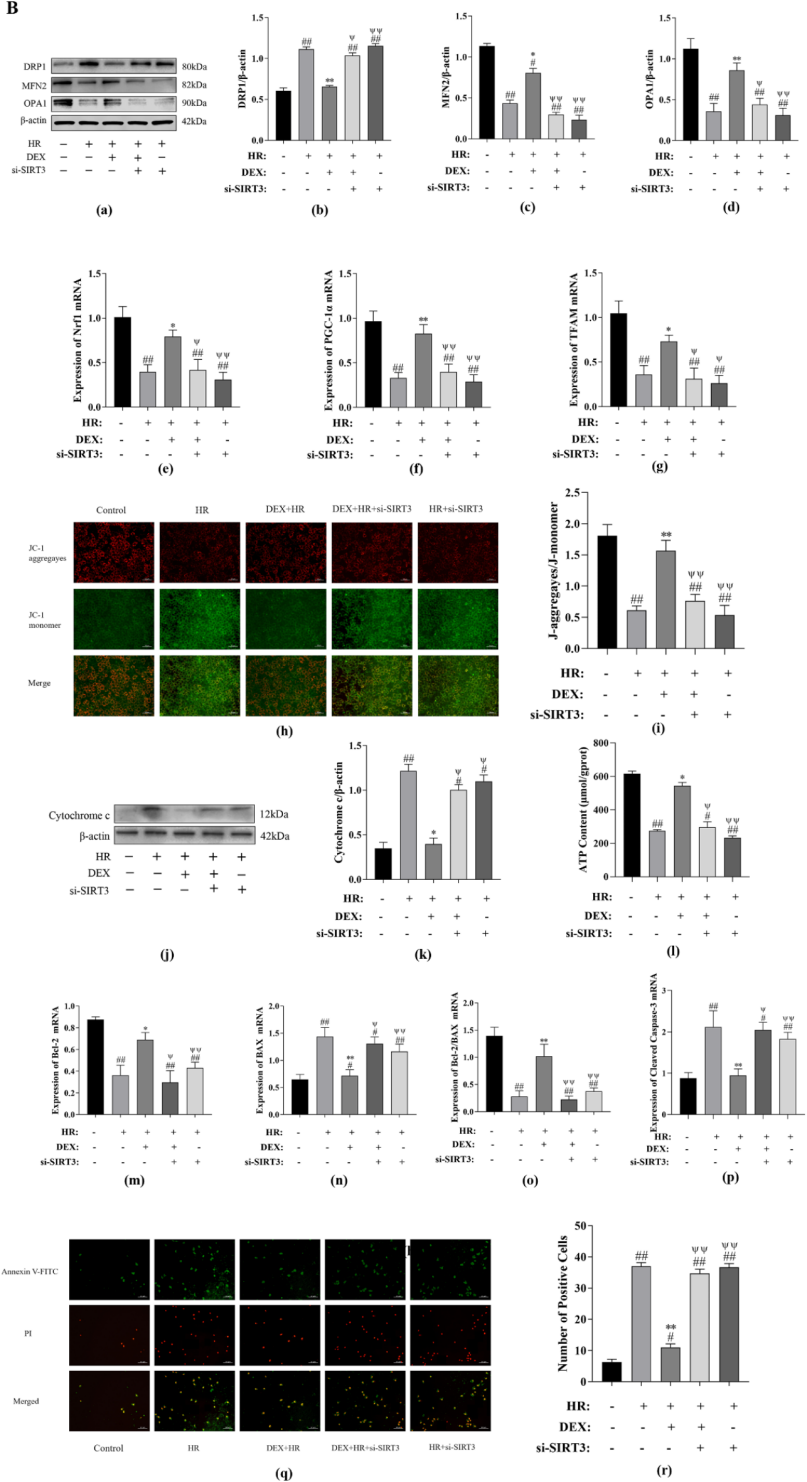
(**A**) Through SIRT3, DEX lessens the damage caused by hypoxia reoxygenation. (**a**) DEX’s results on HR induced SIRT3 protein expression were evaluated by Western-blotting technique. (**b**) The expression of SIRT3 protein. (**c**) Screening of SIRT3 interfering primers. Transfection of SIRT3_1, si-SIRT3_2, or si-NC into BRL-3 A cells was done. The levels of SIRT3 mRNA expression were evaluated using qRT-PCR. (**d**) DEX enhances cell viability through SIRT3. (**e**–**h**) DEX reverses the expression of HR induced oxidative activity index through SIRT3. (**i**) Following the silencing of the SIRT3 gene, the impact of DEX on SIRT3 protein expression were evaluated by Western-blotting technique. (**j**) The expression of SIRT3 protein. All data are presented as mean ± SD (*n* = 3). Compared with the control group, ^##^*p* < 0.01, ^#^*p* < 0.05; compared with the HR group, ^**^p < 0.01, ^*^p < 0.05; compared with the DEX + HR group, ^ψψ^*p* < 0.01, ^ψ^*p* < 0.05. (**B**) Through SIRT3, DEX controls mitochondrial fusion and division, regulates mitochondrial biogenesis, decrease mitochondrial damage and reduces cell apoptosis. (**a**) Representative blots. (**b**) DRP1 protein expression. (**c**) MFN2 protein expression. (**d**) OPA1 protein expression. (**e**) Nrf1 mRNA expression. (**f**) PGC-1 mRNA expression. (**g**) TFAM mRNA expression. (**h**) the mitochondrial membrane potential was measured using the JC-1 assay (scale bar: 50 μm). (**i**) the quantitative analysis was reported as the ratio of JC-1 aggregates to monomers. (**j**) Representative blots. (**k**) cytochrome C expression. (**l**)The proportional amount of ATP. (**m**) Bcl-2 expression. (**n**) BAX expression. (**o**) Bcl-2/BAX. (**p**) Cleaved Caspase-3 expression. (**q**) By staining with Annexin V-FITC and PI, apoptosis was discovered (scale bar = 200 μm). (**r**) Quantification of apoptotic cells. All data are presented as mean ± SD (*n* = 3). Compared with the control group, ^##^*p* < 0.01, ^#^*p* < 0.05; compared with the HR group, ^**^p < 0.01, ^*^p < 0.05; compared with the DEX + HR group, ^ψψ^*p* < 0.01, ^ψ^*p* < 0.05.

As shown in Fig. 5A(a, b), We first looked at how DEX affected the amount of SIRT3 expression. While the DEX group’s SIRT3 expression level did not significantly differ from the control group (*P* > 0.05), the HR group’s SIRT3 expression level was markedly lower (*P* < 0.01). However, the DEX + HR group enhanced the level of SIRT3 expression as opposed to the HR group (*P* < 0.05). To investigate if SIRT3 is involved in DEX’s ability to protect BRL-3 A cells from hypoxia reoxygenation harm, our team transfected si-SIRT3_1, si-SIRT3_2 or si-NC into BRL-3 A cells, and qRT-PCR was used to determine the transfection effectiveness. The si-SIRT3_ 2 group’s expression of SIRT3 was considerably lower than that of the si-NC group (*P* < 0.01) (Fig. 5A(c)). Subsequently, si-SIRT3_ 2 was used for gene silencing related experiments. After SIRT3 gene silencing, we found signs of oxidative stress, cell viability, and SIRT3 protein expression. According to Fig. 5A(d), when the SIRT3 gene is silenced, the ability of DEX to enhance cell viability is reversed (*P* < 0.05). Figure 5A(e–h) illustrates, compared to the HR group, DEX increased the expression of GSH-PX and T-AOC and restored the oxidative enzyme system (*P* < 0.05 or *P* < 0.01); however, si-SIRT3 partially impaired the anti-oxidant enzyme system’s function (*P* < 0.05). Furthermore, DEX reduces NO and H_2_O_2_ expression is also reversed by SIRT3 (*P* < 0.05 or *P* < 0.01). As shown in Fig. 5A(i, j), when the SIRT3 gene is silenced, the ability of DEX to increase SIRT3 protein expression is also reversed (*P* < 0.05).

After then, we looked more closely at how inhibiting SIRT3 affected DEX to mitochondrial quality control system in BRL-3 A cells. By using si-SIRT3, DEX-induced changes to the expression of DRP1, MFN2, and OPA1 were reversed (*P* < 0.05 or *P* < 0.01) (Fig. 5B(a–d)). Additionally, the DEX-restored levels of mRNA expression of the mitochondrial biogenic markers Nrf1, PGC-1, and TFAM were drastically decreased by si-SIRT3 (*P* < 0.05 or *P* < 0.01) (Fig. 5B(e–g)). We silenced SIRT3 to investigate whether DEX enhances mitochondrial activity in BRL-3 A cells is associated with the SIRT3 gene. As depicted in Fig. 5B(h, i,l), DEX therapy mitigated the effects of HR injure, which damaged mitochondrial membrane potential and reduced mitochondrial ATP (*P* < 0.05 or *P* < 0.01). SIRT3 downregulation negated the protective effects of DEX on these elements (*P* < 0.05 or *P* < 0.01). At last, we investigated the potential for SIRT3 knockdown to inhibit DEX’s anti-apoptotic action on HR damage in vitro. According to Fig. 5B(j, k,m–r), by raising the expression of Cleaved Caspase-3, Cytochrome c, BAX, and the amount of apoptotic cells, SIRT3 knockdown could eliminate the anti-apoptotic action of DEX (*P* < 0.05 or *P* < 0.01), decreasing the expression of Bcl-2 and Bcl-2/BAX (*P* < 0.05 or *P* < 0.01). Accordingly, these findings demonstrated that DEX reduces oxidative stress, improves mitochondrial quality control system, increases SIRT3 expression, maintains mitochondrial function, and lowers cell apoptosis.

### DEX exerts protective effect of liver IR injury in vivo

**Fig. 6:**
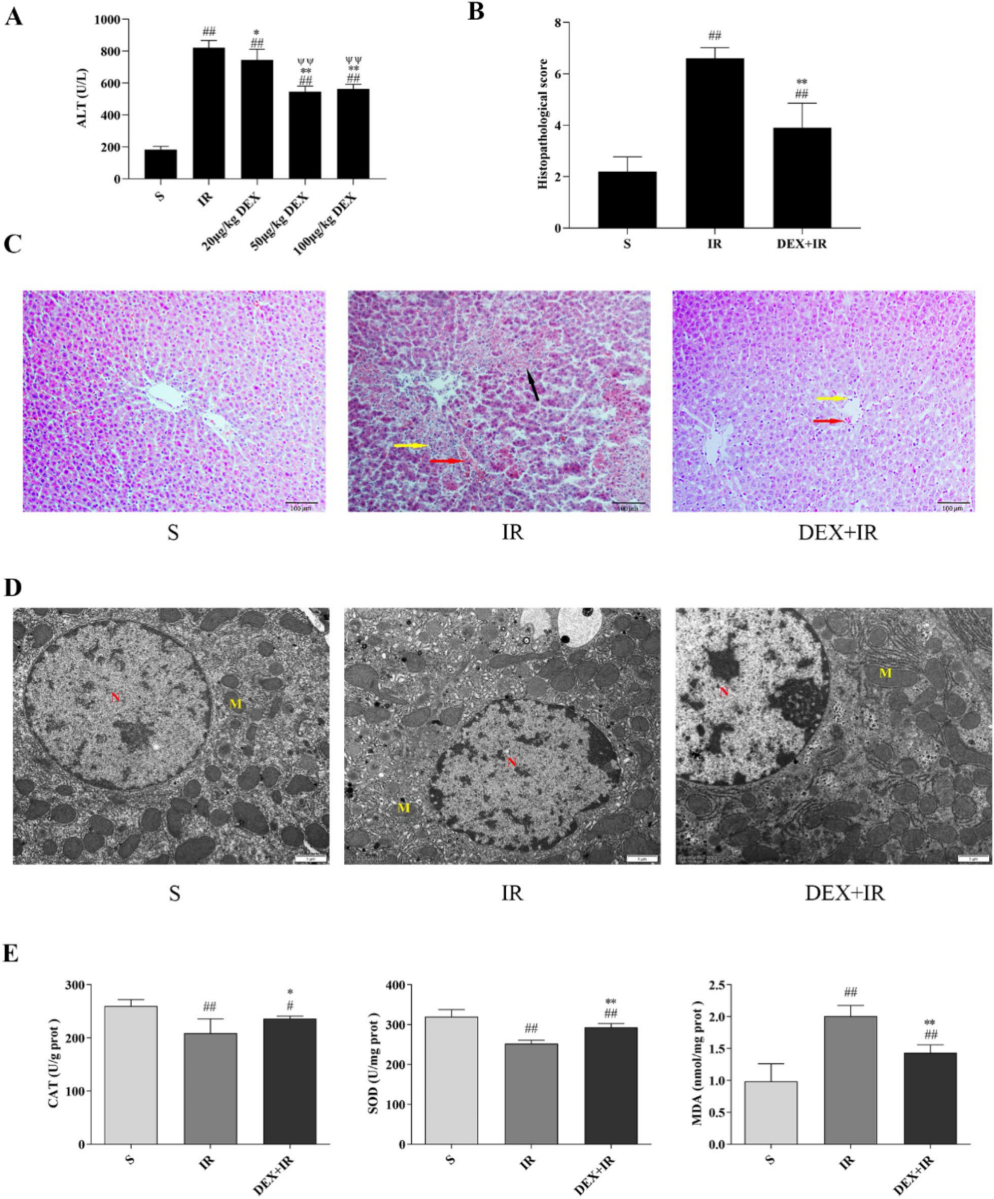
In vivo liver IR injury is protected against by DEX. (**A**) Serum ALT levels in mice. (**B**) Histopathological mean liver injury scores. (**C**) DEX’s effects on liver histopathology brought on by IR. Hepatocyte degeneration necrosis is indicated by the black arrows, hemorrhage by the red arrows, and inflammation by the yellow arrows. (Magnification 200×, scale bars = 100 μm). (**D**) DEX’s effects on hepatocytes’ morphological alterations brought on by IR. N (nucleus) and M (mitochondria) (Magnification 15000×, scale bar = 1 μm). (**E**) DEX lessens IRI-induced hepatic oxidative damage. All data are presented as mean ± SD(*n* = 6). Compared with the sham group, ^##^*p* < 0.01, ^#^*p* < 0.05; compared with the IR group, ^**^p < 0.01, ^*^p < 0.05; compared with the 20 µg/kg DEX group, ^ψψ^*p* < 0.01.

The sham group’s serum ALT activity was 189.0 ± 16.4 U/L. Hepatic IR elevated blood ALT activity to 802.2 ± 14.1 U/L, and the entire DEX-treated group reduced this rise (Fig. 6A). Notability, compared to the 20 µg/kg DEX group, serum ALT activity was considerably lower in the 50 µg/kg and 100 µg/kg DEX groups (*P* < 0.01); the serum ALT activity in the 50 µg/kg DEX group and the 100 µg/kg DEX group did not differ substantially from one another (*P >* 0.05). As a result, we decided that 50 µg/kg of DEX was the optimal pharmacological dose for further investigation. As depicted in Fig. 6C, the hepatocytes in the sham group were well-structured and neatly arranged. However, the microstructure of the liver was damaged in the IR group, including intrahepatic hemorrhage, hepatocyte degeneration necrosis, and hepatocyte inflammatory. These histological pathologies were attenuated by DEX. In Fig. 6B, the histopathological scoring was displayed. The IR group’s liver tissue damage score was substantially higher than that of the DEX + IR and sham groups (*P* < 0.01). The liver damage score of the DEX + IR group following DEX pretreatment was considerably less than that of the IR group (*P* < 0.01). Figure 6D depicted the ultrastructure of hepatocytes. The sham group displayed normal histological structure, including distinct nuclei, integral nuclear membranes, uniform distribution of nucleoplasm, and many mitochondria. When compared, abnormal mitochondrial structure (mitochondrial swelling and dissolution), crumpled nuclei and increased nuclear pores were observed in the IR group. Comparing the DEX + IR group to the IR group, abnormal changes in mitochondria and nuclei have been improved. According to Fig. 6E, In comparison to the sham group, the MDA content in the IR group considerably increased (*P* < 0.01). In contrast, treatment with DEX reduced the rise in MDA levels (*P* < 0.01). Meanwhile, when opposed to the sham group, SOD and CAT activity was dramatically reduced in the IR group (*P* < 0.01). Furthermore, Comparing the DEX + IR group to the IR group, SOD and CAT activities were considerably higher in the DEX + IR group (*P* < 0.01 or *P* < 0.05). DEX reduced the harm on both organelles and nuclei and DEX can alleviate the IR- induced hepatic oxidative burden.

### DEX reduce mitochondrial damage, apoptosis, regulate mitochondrial homeostasis and the expression of SIRT3 during liver IR injury in vivo

**Fig. 7:**
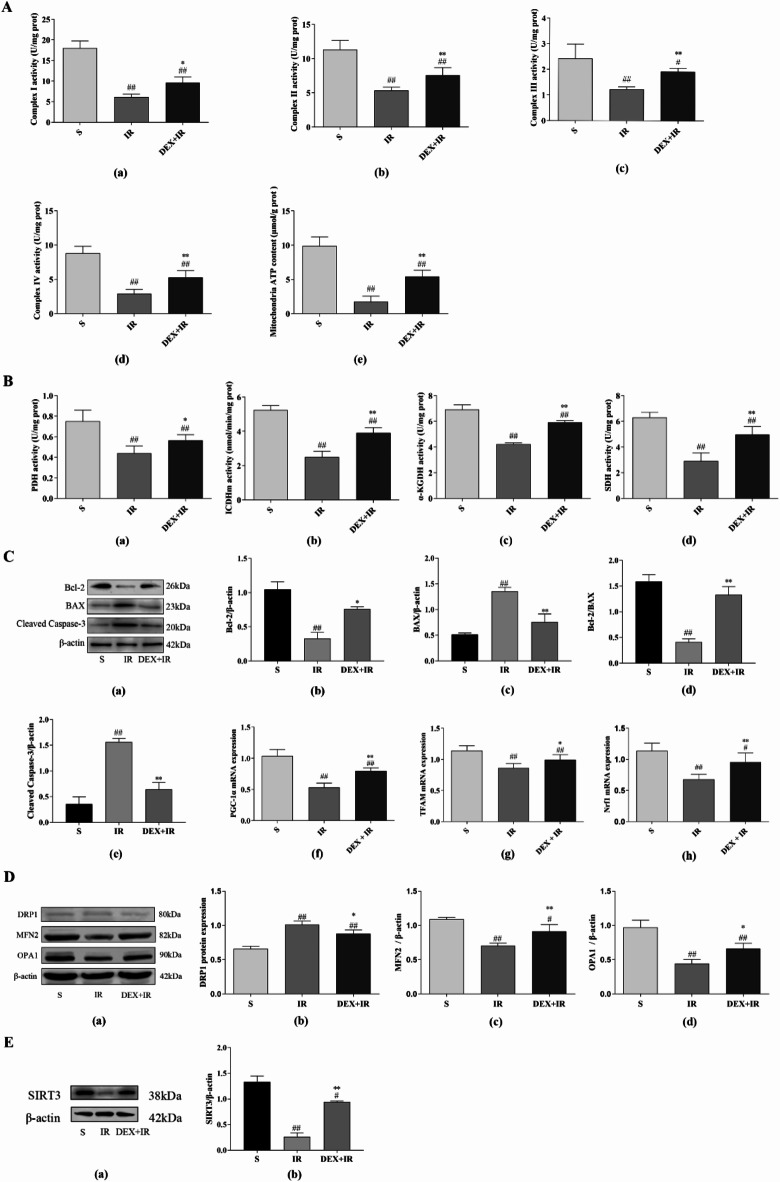
DEX reduce mitochondrial damage, regulates mitochondrial homeostasis and SIRT3 expression during in vivo liver IR injury. (**A**(**a**–**d**)) Complexes I, II, III, and IV of the respiratory chain’s activity. (**e**) Total ATP content. (*n* = 6). (**B**) Active expression of TCA cycle enzymes (*n* = 6). (**C**(**a**–**e**)) Cleaved caspase-3, BAX, and Bcl-2 protein expression was assessed using a western blotting test (*n* = 3). (**C**(**f**–**h**)) In the rat liver, TFAM, Nrf1, and PGC-1 are expressed (*n* = 6). (D) DRP1, MFN2, and OPA1 protein expression was evaluated using a western blotting technique (*n* = 3). (**E**) SIRT3 protein expression was evaluated using a western blotting technique (*n* = 3). All data are presented as mean ± SD. Compared with the sham group, ^##^*p* < 0.01, ^#^*p* < 0.05; compared with the IR group, ^**^p < 0.01, ^*^p < 0.05.

As depicted in Fig. 7A(a–d), the intricate I, II, III, and IV actions in the sham group were 18.67 ± 3.40 U/mg prot, 11.32 ± 1.34 U/mg prot, 2.42 ± 0.56 U/mg prot, and 8.80 ± 1.02 U/mg prot respectively, all of which in the IR group were markedly reduced to 33, 47, 51 and 33% (*P* < 0.01), respectively, and DEX attenuated this decrease (*P* < 0.01 or *P* < 0.05). Meanwhile, as depicted in Fig. 7A(e) 18.68 ± 2.31 µmol/g prot was the hepatic ATP concentration in the sham group. This level was reduced by hepatic IR to 33% of the sham group (*P* < 0.01), and DEX mitigated this effect (*P* < 0.01). Additionally, as depicted in Fig. 7B, PDH, -KGDH, SDH, and ICDHm activities in the IR group were markedly lower in comparison to those in the sham group (*P* < 0.01). Notably, the DEX + IR group significantly outperformed the IR group in terms of the activity of every TCA cycle enzyme (*P* < 0.01 or *P* < 0.05). We assessed values of the mitochondria-dependent apoptotic proteins BAX, Bcl-2, and Cleaved caspase-3 to support the protective role of DEX in IR-induced hepatocyte death. According to our findings, liver IR elevated levels of Cleaved Caspase-3 and BAX while lowering levels of Bcl-2 and Bcl-2/BAX in comparison to the sham group (*P* < 0.01), although DEX therapy largely reduced these effects (*P* < 0.01 or *P* < 0.05) (Fig. 7C(a–e)). These findings imply that DEX prevented liver mitochondrion-dependent apoptosis. As shown in Fig. 7C(f–h), PGC-1, Nrf1, and TFAM mRNA expression levels in the IR group were considerably lower than those in the sham group (*P* < 0.01), whereas PGC-1, Nrf1, and TFAM mRNA expression levels in the DEX + IR group were slightly higher than those in the IR group (*P* < 0.01 or *P* < 0.05). The findings showed that hepatic IR caused a malfunction in mitochondrial biogenesis whereas DEX activated it. OPA1 and MFN2 protein expression levels in IR-induced rats were lower than those in sham-operated rats (Fig. 7D; *P* < 0.01), and IR increased the protein expression level of DRP1 in the liver (*P* < 0.01). In contrast, OPA1 and MFN2 protein contents were considerably higher in the DEX + IR group compared to the IR group (*P* < 0.01 or *P* < 0.05), whereas DRP1 protein contents were significantly lower in the DEX + IR group compared to the IR group (*P* < 0.05). The findings showed that liver IR increased mitochondrial fission while decreasing mitochondrial fusion, resulting in an imbalance in mitochondrial dynamics, DEX alleviated these IR-induced changes. SIRT3 protein expression levels in IR-induced rats were considerably lower than in sham rats, as seen in Fig. 7E (*P* < 0.01). In contrast, comparing the DEX + IR group to the IR group, the protein content of SIRT3 in the DEX + IR group was considerably higher (*P* < 0.01). These data imply that DEX can reduce mitochondrial damage, apoptosis, regulate mitochondrial homeostasis and the expression of SIRT3.

## Discussion

Hepatic IR injury is regarded as a typical pathological condition connected to trauma and shock. Numerous investigations on IR injury have demonstrated that mitochondrial functional and structural abnormalities brought on by oxidative stress play a major factor in the damage of hepatocytes during ischemia^35^. Restoring the depleted mitochondrial membrane potential, and reducing the ROS in the mitochondria, DEX is crucial for maintaining mitochondrial homeostasis^36,37^. Recent research has shown that SIRT3 is essential for defending liver cells in opposition to oxidative stress. This protective mechanism is accomplished through enhanced elimination of reactive oxygen compounds and preservation of mitochondrial integrity^38^. Furthermore, DEX alleviates renal IR injury by activating the expression of SIRT3^39^. The findings of the present research indicate that DEX may lessen liver IR damage via altering SIRT3’s expression in mitochondrial quality control.

Liver IR can bring a strong oxidative stress response. In the course of ischemia, oxidative stress equilibrium is crucial. Hepatic IR injury can lead to excessive oxidative stress and finally affect liver structure^39^. Numerous clinical and fundamental studies have shown that DEX has the power to control the body’s equilibrium of oxidative stress and to preserve the liver. DEX has been discovered to lessen kidney and liver harm by decreasing HO-1 and enhancing SOD activity^40^. DEX can cut down on oxidative stress responses and lessen liver damage in the rat liver IR model by boosting the activity of SOD, CAT, and GSH-PX^41^. Our recent findings show that DEX therapy successfully counteracted the rise in ROS, NO, and H_2_O_2_ levels brought on by HR. Additionally, we noted that the decline in GSH-PX and T-AOC activity was also reversed. Consistently, similar results were obtained in our in vivo experiments where DEX treatment reversed the decrease in SOD and CAT activity and the rise in MDA content. These findings strongly suggest that DEX has the potential to ameliorate liver injury induced by IR through its ability to reduce oxidative damage.

Mitochondria serve as crucial cell organelles that are where energy is produced. The onset and progression of IR injury are significantly influenced by mitochondrial dysfunction^42^. Cell damage can cause a decrease in MMP and hinder mitochondrial ATP synthesis^43^. DEX is crucial in the treatment of mitochondrial dysfunction^44,45^. DEX has effects in rats with severe lung damage by preventing cellular ATP levels and mitochondrial transmembrane potential from falling^46^. Via opening ATP channels in the mitochondria, Yuan et al. demonstrated that rats may be protected by DEX from focal cerebral IR injury^45^. Our findings are similar with recent studies, which found that liver IR injury reduced MMP and ATP content, as well as the changes were mitigated by DEX. The electron transport chain (ETC) is the key player in mitochondrial energy production^47^. Reduced mitochondrial ATP production, which occurs in liver IR injury, can be caused by a lack of electrons or by the ETC being blocked^48^. Following orthotopic liver transplantation, ischemia damage was associated with malfunctioning of complexes II and III, decreased complex I activity, and increased ROS production^49,50^. In our investigation, hepatic IR reduced complexes I–IV activity, while DEX partially recovered it. The primary processes for lipid, protein, and carbohydrate metabolism as well as the generation of energy in mitochondria are called TCA cycles. Different acute and chronic liver diseases entail a reduction in the activity of TCA cycle enzymes. However, whether DEX rescued TCA cycle function induced by liver IR is unclear. In our investigation, DEX therapy improved the alterations caused by IR on the hepatic PDH, ICDHm, α-KGDH, and SDH activities in mice. These findings suggested that DEX can lessen hepatic IR-induced deficits of mitochondrial TCA cycle functioning. Besides that DEX can enhance mitochondrial function by rescuing mitochondrial complexes I to IV, MMP, and ATP during hepatic IR.

Control internal or mitochondrial apoptotic pathways are Bcl-2 and BAX^51^. BAX is primarily located in the cytoplasm of healthy cells. Bcl-2 stops the outer mitochondrial membrane from deteriorating, hence preventing cell apoptosis^52^. Apoptosis of cells is thought to be influenced by a decline in the Bcl-2/BAX ratio, which initiates caspase-3-mediated apoptosis^53^. Cytochrome c translocation is a necessary component of the apoptotic process^54^. Caspase-3 is activated by cytochrome c, and this eventually causes cell death^55^. Cytochrome c, BAX, and Bcl-2 are released in large amounts as a result of stress brought on by anesthesia and surgery, according to studies, which upsets the harmony between the creation of ROS and antioxidant defense systems^56,57^. However DEX treatment decreased the expression of BAX in intestinal tissue while increasing Bcl-2 and Cleaved Caspase-3 expression, indicating its preventive and therapeutic effects on intestinal IR injury^58^. In our study, Bcl-2/BAX protein expression was downregulated, but Cleaved Caspase-3 and Cytochrome c protein levels were upregulated in HR-damaged liver cells. Apoptosis-positive cells also became more prevalent. Bcl-2/BAX protein expression rose after pretreatment with DEX, but Cleaved caspase-3 and Cytochrome c expression dropped and the proportion of apoptotic cells increased. This finding was confirmed in vivo which suggest that DEX alleviates liver IR injury by inhibiting internal mitochondria-dependent apoptosis.

Mitochondrial quality control (MQC) is a system that eukaryotic cells have evolved to monitor mitochondrial health and activate appropriate responses, integrating mitochondrial biogenesis and dynamics, maintaining mitochondrial health and speedily removing or repairing damaged mitochondria^59^. Mitochondria, which are active organelles, continuously go through fission and fusion processes to meet cellular energy needs^60,61^. However, excessive mitochondrial fission after cardiac IR injury led to an imbalance in mitochondrial homeostasis and heart malfunction^62,63^. In a cerebral artery blockage model, Zhang et al. showed that the intrinsic apoptotic pathway required enhanced mitochondrial fission^64^. Considering the earlier investigations, we discovered that hepatic IR injury increased the expression of the DRP1 protein which is the central mediator of mitochondrial fission, and DEX can down-regulate the increase both in vivo and in vitro. From this, we know that DEX inhibited mitochondrial fission produced by hepatic IR damage. Mitochondrial fusion is a membrane-anchored MFN2 and OPA1-dependent process^65^. Hepatic IR injury markedly decreased MFN2 protein level^66^, and hepatic deletion of MFN2 induced ROS overproduction^35^. In the current work, hepatic HR damage reduced MFN2 and OPA1 protein expression, and DEX mitigated these alterations, which were also supported by in vivo tests. According to our research, DEX controls the imbalance in mitochondrial dynamics brought on by IR by lowering fission and increasing fusion.

By increasing the amount of mitochondria, mitochondrial biogenesis meets the energy requirements of cells with damaged or defective mitochondria^67^. The PGC-1-Nrf1-TFAM pathway is closely regulated in terms of mitochondrial biogenesis. PGC-1 activation causes Nrf1, which triggersgenetic transcription. TFAM is activated by Nrf1 to promote transcription of mitochondrial genes and genome replication^68^. In order to offset the ATP deficiency in hypoxia and ischemia, maintaining early-stage mitochondrial content is important. However, prolonged ischemia causes PGC-1 and TFAM to decline, which impairs mitochondrial biogenesis^69^. Hepatic IR injury has been treated using a technique that encourages mitochondrial biogenesis^70^. Our research showed that liver ischemia-reperfusion (IR) injury impairs mitochondrial biogenesis, as seen by a decrease in Nrf1, PGC-1, and TFAM. However, our results showed that in vivo and in vitro, DEX stimulates Nrf1, PGC-1, and TFAM expression, which in turn increases mitochondrial biogenesis. These outcomes strongly suggest that DEX effectively mitigates IR injury by promoting the process of mitochondrial biogenesis.

In order to investigate whether DEX can improve mitochondrial function and mitochondria-dependent apoptosis through the mitochondrial quality control system, We treated BRL-3 A cells with Mdivi-1, a mitochondrial fission inhibitor, and SR-18,292, an inhibitor of mitochondrial biogenesis. SR-18,292 increases the interaction between PGC-1α and acetyltransferase GCN5, and reduces the activation of nuclear hormone receptor HNF4α by PGC-1α^30^. Mdivi-1, as a mitochondrial inhibitor, can inhibit Drp1 mediated excessive mitochondrial division, reduce the release of Cytc from dividing mitochondria, alleviate cell apoptosis, and inhibit the secretion of inflammatory factors, thereby improving organ damage^31^. The results demonstrated that Mdivi-1 improves mitochondrial function related factor content and regulate apoptosis related protein expression. Mdivi-1 and DEX both inhibit IR-induced mitochondrial dysfunction and apoptosis. SR-18,292, on the other hand, reversed the negative impact of DEX on mitochondrial activity and apoptosis. All of these demonstrate how DEX, which inhibits mitochondrial fission and encourages mitochondrial biogenesis, can enhance mitochondrial function and control mitochondria-dependent apoptosis.

It’s interesting to note that our research revealed a potential link between the stimulation of the SIRT3 pathway and the mechanism through which DEX delivers its effects. SIRT3 primarily localizes in the mitochondrial matrix and serves as a significant deacetylase functions as a key mitochondrial stress response protein and participates in the regulation of mitochondrial activity^71^. According to numerous studies, SIRT3 is essential for maintaining mitochondrial homeostasis and controlling ROS^72,73^. In IR injury, SIRT3 has a protective role as well. According to one study, SIRT3 knockdown animals had lower mitochondrial function and were more vulnerable to IR injury^74^. However, after IR injury, SIRT3 overexpression enhanced mitochondrial fusion and reduced renal failure^75^. Studies have confirmed that DEX has the ability to control SIRT3 expression. DEX guards neurons from IR damage by regulating SIRT3^76^. Furthermore, DEX could attenuate hepatic IR-induced hippocampal damage and cognitive dysfunction by increasing SIRT3 expression and activity^77^. As a result of liver IR injury, SIRT3 expression was consistently observed to be markedly decreased in our study. In contrast, SIRT3 expression was markedly elevated by DEX both in vivo and in vitro. In BRL-3 A cells, silencing SIRT3 prevented DEX from improving mitochondrial function and kinetics while also accelerating oxidative stress and cell death. This shows that the SIRT3 pathway’s activation may be necessary for DEX to reduce mitochondrial damage and maintain mitochondrial homeostasis in order to mitigate liver IR harm.

## Conclusion

In summary, we show that DEX therapy protect mitochondrial damage, which considerably lessens liver IR harm. DEX’s protection against liver IR injury is mediated by the modulation of mitochondrial quality control. Additionally, the regulation of SIRT3 activity could be responsible for this outcome. These results might point to a useful therapeutic target in the liver IR injury etiology.

It must be highlighted, nevertheless, that there are still a lot of issues to be resolved and that it is still unclear what the underlying pathogenic pathways are that cause liver IR harm. Consequently, we can continue to explore the specific signaling pathway through which DEX acts on SIRT3.

## Electronic supplementary material

Below is the link to the electronic supplementary material.

Supplementary Material 1

## Data Availability

The datasets analyzed during the current study are not publicly available but are available from the corresponding author on reasonable request.

## Abbreviations

IR: Ischaemia-reperfusion
SIRT3: Sirtuin 3
DEX: Dexmedetomidine
HR: Hypoxia-reoxygenation
ATP: Adenosine triphosphate
ETC: Electron transport chain
ROS: Reactive oxygen species
Nrf1: Nuclear respiratory factor 1
PGC-1α: Peroxisome proliferator-activated receptor γ coactivator 1α
TFAM: Mitochondrial transcription factor A
MFN: Mitofusin
OPA1: Optic atrophy 1
DRP1: Dynamin-related protein 1
Fis1: Fission protein 1
GSH-PX: Glutathione peroxidase
T-AOC: Total antioxidant capacity
MMP: Mitochondrial membrane potential
ALT: Alanine aminotransferase
MDA: Malondialdehyde
SOD: Superoxide dismutase
CAT: Catalase
PDH: Pyruvate dehydrogenase
α-KGDH: α-ketoglutarate dehydrogenase
ICDHm: Succinic dehydroge and isocitric dehydrogenase
TCA: Tricarboxylic acid
Bcl-2: B-cell lymphoma-2
BAX: Bcl-2-associated X protein
MQC: Mitochondrial quality control
