# Supplementary material for: Dexmedetomidine ameliorates hepatic ischemia reperfusion injury via modulating SIRT3 mediated mitochondrial quality control
Source: Sci Rep. 2025 Feb 15;15:5630. doi: 10.1038/s41598-025-90069-1 (PMC11829960; doi:10.1038/s41598-025-90069-1)
Supplement: Supplementary file 1 — Supplementary Material 1 [file 41598_2025_90069_MOESM1_ESM.pdf]

Figure 3

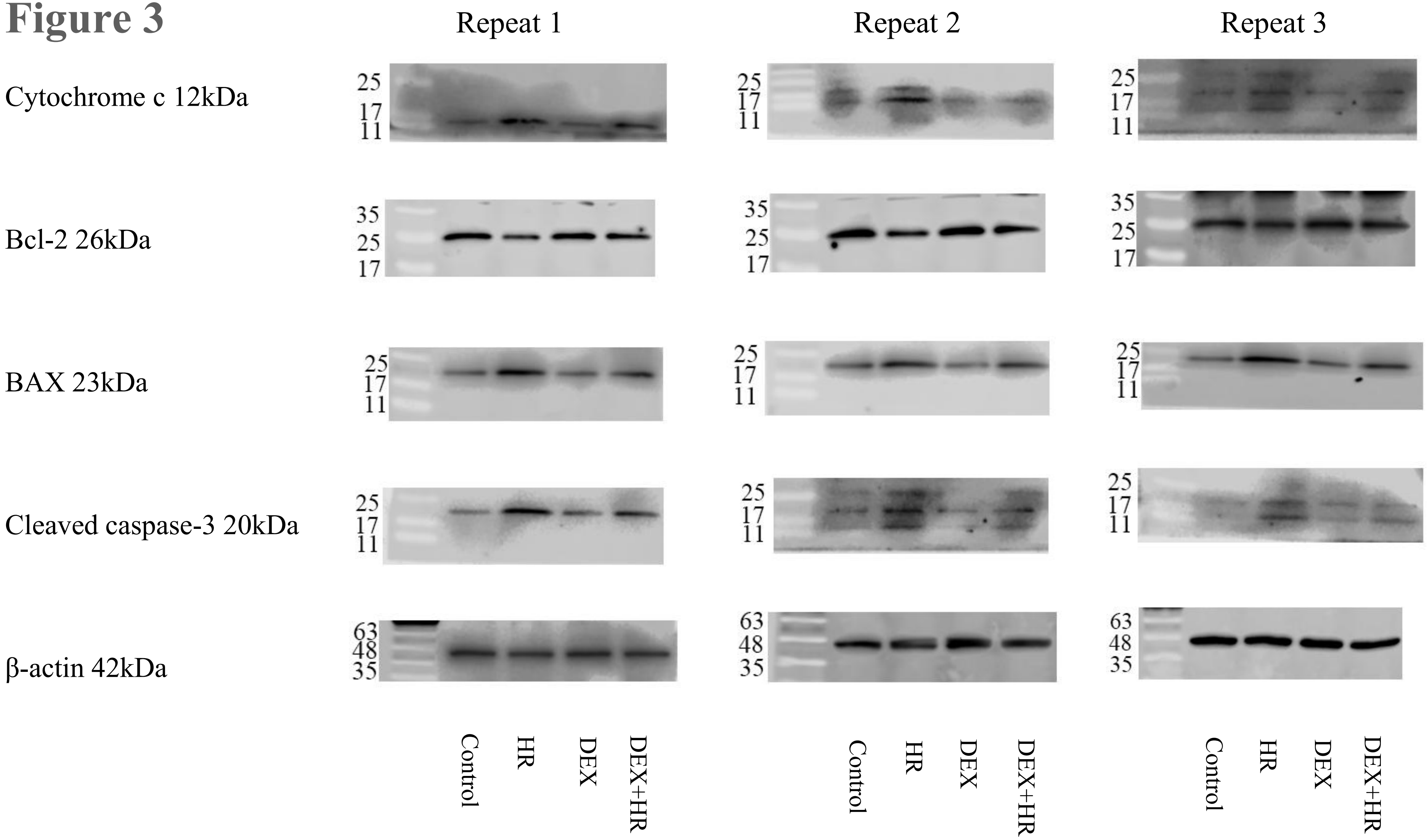

Repeat 1
Repeat 2
Repeat 3
Cytochrome c 12kDa
Bcl-2 26kDa
BAX 23kDa
Cleaved caspase-3 20kDa
β-actin 42kDa
25
17
11
35
63
48
Control
HR
DEX
DEX+HR

Figure 4

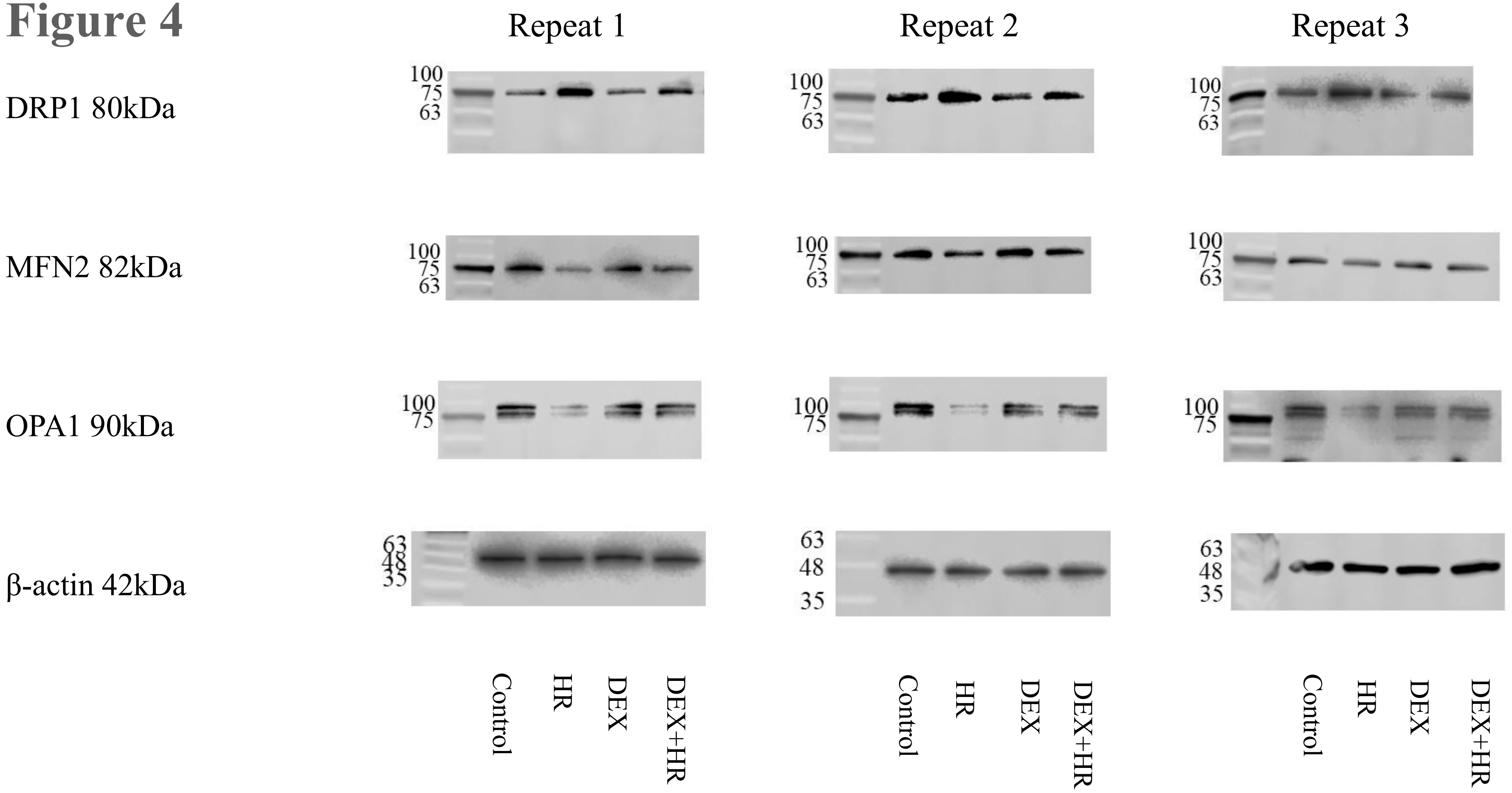

Repeat 1
Repeat 2
Repeat 3
DRP1 80kDa
MFN2 82kDa
OPA1 90kDa
β-actin 42kDa
100
75
63
48
35
Control
HR
DEX
DEX+HR

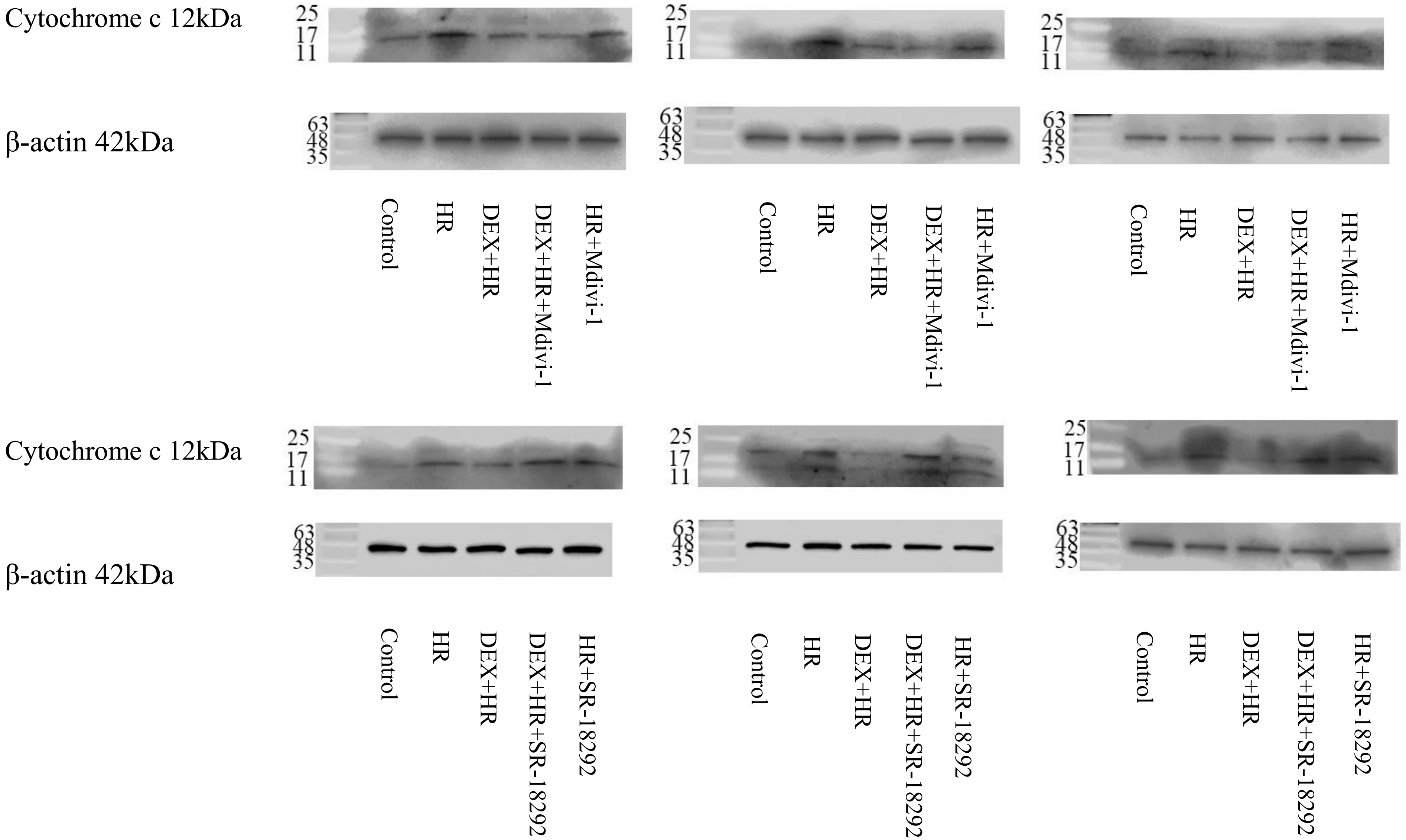

Cytochrome c 12kDa
β-actin 42kDa
25
17
11
63
48
35
Control
HR
DEX+HR
DEX+HR+Mdivi-1
HR+Mdivi-1
Cytochrome c 12kDa
β-actin 42kDa
Control
HR
DEX+HR
DEX+HR+SR-18292
HR+SR-18292

Figure 5

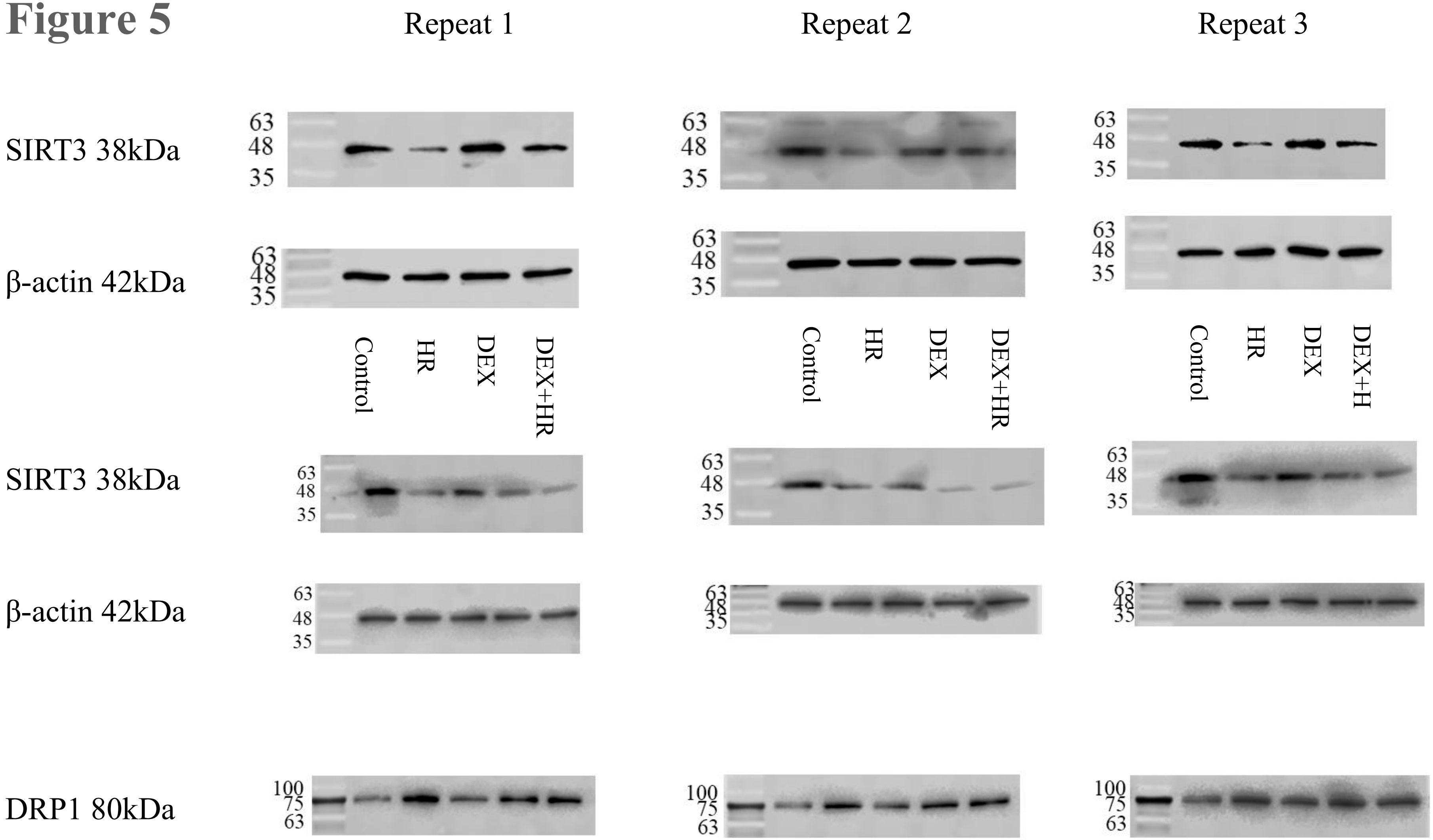

Repeat 1
Repeat 2
Repeat 3
SIRT3 38kDa
β-actin 42kDa
Control
HR
DEX
DEX+HR
DEX+H
SIRT3 38kDa
β-actin 42kDa
DRP1 80kDa
63
48
35
100
75

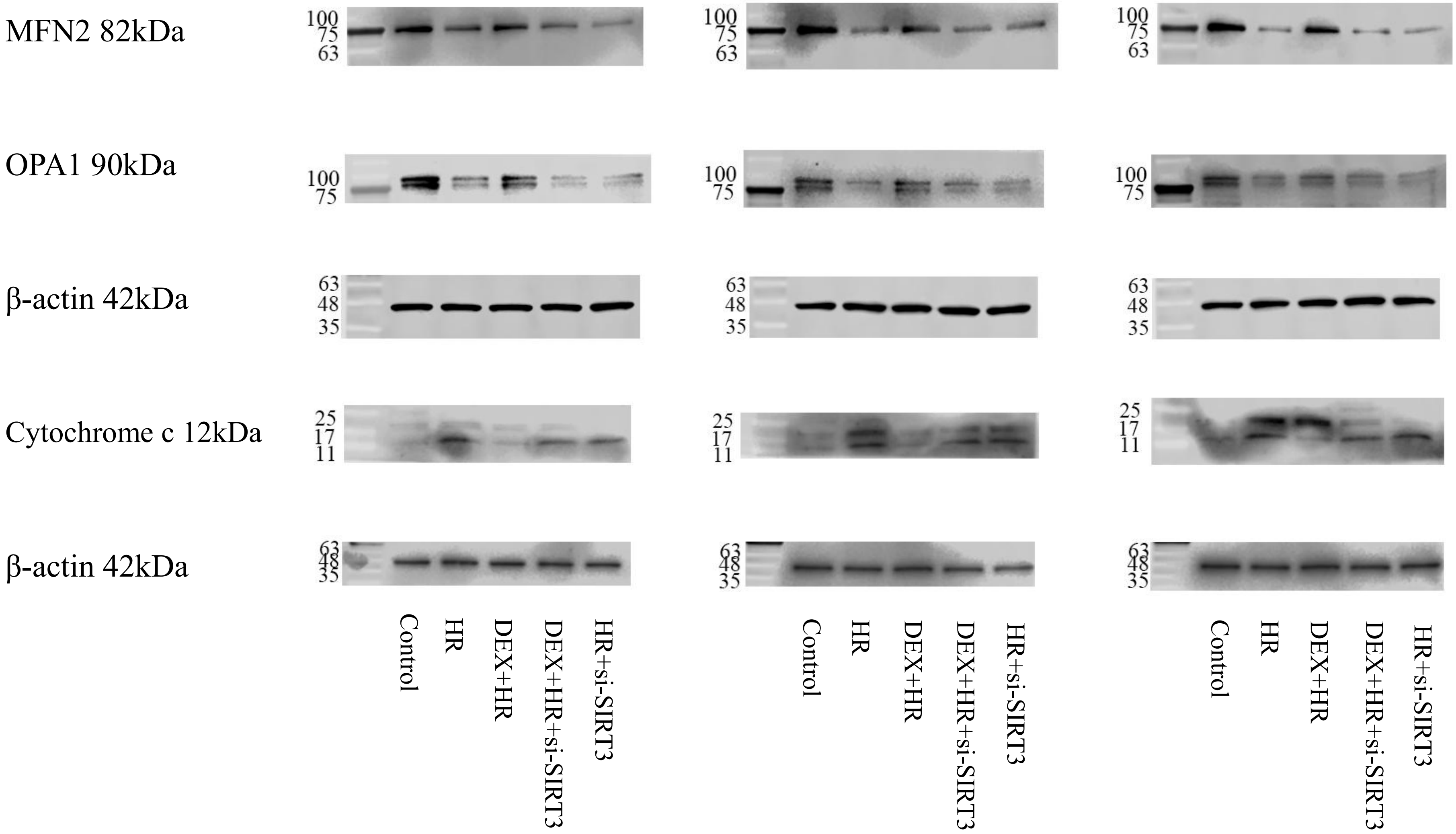
MFN2 82kDa
OPA1 90kDa
β-actin 42kDa
Cytochrome c 12kDa
β-actin 42kDa
Control
HR
DEX+HR
DEX+HR+si-SIRT3
HR+si-SIRT3

Figure 7

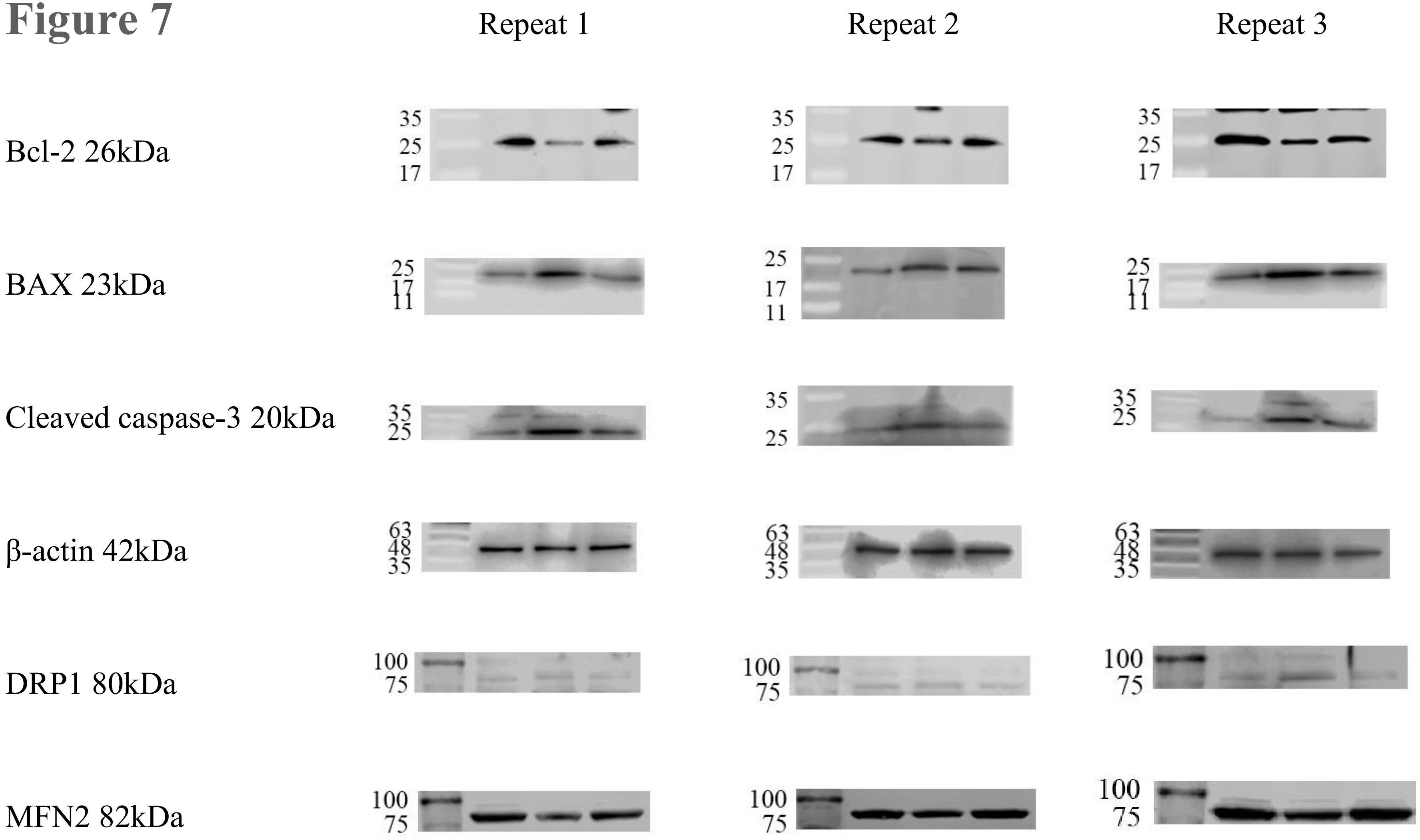

Repeat 1
Repeat 2
Repeat 3
Bcl-2 26kDa
BAX 23kDa
Cleaved caspase-3 20kDa
β-actin 42kDa
DRP1 80kDa
MFN2 82kDa
35
25
17
11
63
48
100
75

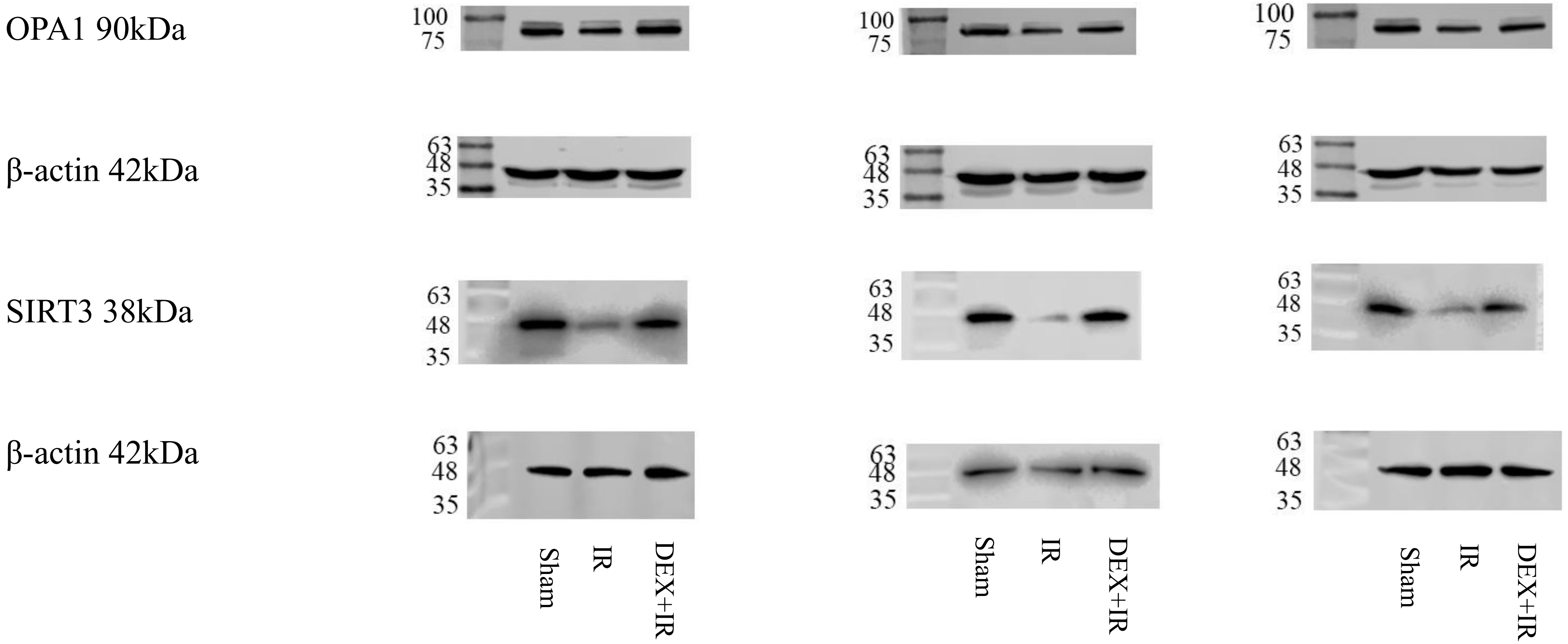

OPA1 90kDa
β-actin 42kDa
SIRT3 38kDa
β-actin 42kDa
100
75
63
48
35
Sham
IR
DEX+IR
